# Multi-Omics Characterization of Circular RNA-Encoded Novel Proteins Associated With Bladder Outlet Obstruction

**DOI:** 10.3389/fcell.2021.772534

**Published:** 2022-01-07

**Authors:** Baoyi Zhu, Zhanfang Kang, Sihua Zhu, Yuying Zhang, Xiangmao Lai, Lilin Zhou, Hai Huang, Xiaofeng Gao, Chonghe Jiang, Jianwen Zeng

**Affiliations:** ^1^ Department of Urology, The Sixth Affiliated Hospital of Guangzhou Medical University, Qingyuan, China; ^2^ Department of Basic Medical Research, The Sixth Affiliated Hospital of Guangzhou Medical University, Qingyuan, China; ^3^ Department of Urology, Sun Yat-sen Memorial Hospital, Sun Yat-sen University, Guangzhou, China; ^4^ Department of Urology, Changhai Hospital, Naval Medical University, Shanghai, China

**Keywords:** circRNA, iTRAQ proteomics, circRNA-encoded protein, protein-encoding circRNA, bladder outlet obstruction

## Abstract

Bladder outlet obstruction (BOO) is a common urologic disease associated with poorly understood molecular mechanisms. This study aimed to investigate the possible involvements of circRNAs (circular RNAs) and circRNA-encoded proteins in BOO development. The rat BOO model was established by the partial bladder outlet obstruction surgery. Differential expression of circRNA and protein profiles were characterized by deep RNA sequencing and iTRAQ quantitative proteomics respectively. Novel proteins encoded by circRNAs were predicted through ORF (open reading frame) selection using the GETORF software and verified by the mass spectrometry in proteomics, combined with the validation of their expressional alterations by quantitative RT-PCR. Totally 3,051 circRNAs were differentially expressed in bladder tissues of rat BOO model with widespread genomic distributions, including 1,414 up-regulated, and 1,637 down-regulated circRNAs. Our following quantitative proteomics revealed significant changes of 85 proteins in rat BOO model, which were enriched in multiple biological processes and signaling pathways such as the PPAR and Wnt pathways. Among them, 21 differentially expressed proteins were predicted to be encoded by circRNAs and showed consistent circRNA and protein levels in rat BOO model. The expression levels of five protein-encoding circRNAs were further validated by quantitative RT-PCR and mass spectrometry. The circRNA and protein profiles were substantially altered in rat BOO model, with great expressional changes of circRNA-encoded novel proteins.

## Introduction

Bladder outlet obstruction (BOO) is a urological condition featured by voiding difficulty, abdominal straining during voiding, increased postvoid residual and reduced urine flow rate due to increased detrusor pressure, and which is usually associated with lower urinary tract symptoms (LUTS) (Meier and Padmanabhan, 2016; Niemczyk et al., 2018). The prevalence of BOO was previously predicted to be as high as 21.8% among population aged over 20 years, which is affecting more than one billion patients worldwide (Niemczyk et al., 2018). It is known that BOO pathogenesis could be induced by anatomic factors including benign prostatic obstruction (BPO), pelvic organ prolapses, stricture diseases, urologic malignancies and iatrogenic obstruction, as well as functional causes such as dysfunctional voiding, primary bladder neck obstruction, and Fowler’s syndrome (King and Goldman, 2014; Meier and Padmanabhan, 2016; Fusco et al., 2018). Neurologic conditions like multiple sclerosis, spinal cord injuries, and or Parkinson’s disease could also lead to BOO pathogenesis (Meier and Padmanabhan, 2016). Recent studies showed that the progression and persistence of BOO usually resulted into various morphological and molecular alterations in the urothelium, such as severe urothelial dysfunctions and cell apoptosis in suburothelium, associated with great changes in signaling pathways and interruption of barrier, transport, and sensory functions in urothelium (Fusco et al., 2018; Niemczyk et al., 2018). Moreover, the hypertrophy of smooth muscle cells (SMCs), elevated accumulation of collagen and elastic fibers in extracellular matrix (ECM), and resultant intrafascicular fibrosis were also frequently observed in detrusor muscles of BOO patients (Fusco et al., 2018). However, the molecular mechanism of BOO pathophysiology is still not well elucidated.

Regulatory non-coding RNAs (ncRNAs) have been well-documented as critical players in various physiological events and pathogenic processes (Matsui and Corey, 2017; Saw et al., 2021). In the ncRNA superfamily, the covalently closed circRNAs in the size of over 100 nt that were produced via the back-splicing of exons in precursor mRNAs, are another essential group of regulatory non-coding RNAs also exerting key effects in disease pathogenesis (Li X. et al., 2018; Saw et al., 2021). Extensive research in the past decade disclosed that CircRNAs performed their biological functions mainly through influencing the transcription and splicing of their parental genes, sponging other miRNAs, or directly binding with functional proteins (Li X. et al., 2018). For instance, the invasion and metastasis of bladder cancer cells was reported to be substantially regulated by the hsa_circ_0001361, via directly sponging the miR-491-5p to promote the expression of matrix metalloproteinase 9 (MMP9) gene (Liu et al., 2020). Moreover, the significance of circRNAs in bladder cancer development were further supported by the investigations of other circRNAs such as circSLC8A1 and bladder cancer related circRNA-3 (BCRC-3) (Li M. et al., 2018; Xie et al., 2018; Lu et al., 2019). In addition, the expression of a large number of circRNAs were recently shown to be significantly changed in the bladder cancer tissues compared with adjacent normal bladder tissues (Li M. et al., 2018). These discoveries indicated the close involvements of circRNAs in bladder physiology and pathogenesis, but little is known about the expression and functions of circRNAs in BOO development and progression.

Besides regulating parental gene expression and acting as microRNA sponging, recent reports have also unveiled another layer of functioning mechanisms of circRNAs, and which could be translated as protein-encoding non-coding RNAs (Li X. et al., 2018). In such way, circRNAs were capable of causing rearrangement of the original genetic information designated by human genome (Li X. et al., 2018). For instance, the open reading frame-containing circ-ZNF609 was shown to be translated into a functional protein in human and murine myoblasts, which was associated with myoblast proliferation regulation during myogenesis (Legnini et al., 2017). The SHPRH-146aa protein translated from the circular RNA circ-SHPRH was also characterized recently as the first circRNA-encoded protein associated with cancer pathogenesis (Begum et al., 2018). In recent years, multiple circular RNAs that could be translated into proteins with essential regulatory roles have been identified in the context of cancer development (Xia et al., 2019; Zheng et al., 2019; Pan et al., 2020; Jiang et al., 2021). Among them, the circFNDC3B-218aa protein encoded by the circRNA circFNDC3B was found to repress colon cancer progression and epithelial-mesenchymal transition (EMT) by modulating the expression of Snail gene (Pan et al., 2020). Another protein translated from the circMAPK1 was also recently characterized as suppressor of gastric cancer progression, which is mediating by its inhibition of MAPK (mitogen-associated protein kinase) signaling cascades (Jiang et al., 2021). However, the existence of protein-encoding circRNAs linked with BOO pathogenesis have never been previously explored.

In this study, we performed a transcriptomic characterization of circular RNAs differentially expressed in the rat BOO model, followed by screening of protein-encoding circRNAs through combination of a quantitative proteomics analysis. These investigations proved the first evidence of circRNA-encoded proteins associated with BOO development and progression, which would provide new insights into the non-coding RNA-mediated BOO pathogenesis by further functional explorations.

## Material and Methods

### Animal Model Establishment

The modeling of BOO in rats was finished by partial bladder outlet obstruction surgery as previously described (Majima et al., 2020). Briefly, female Sprague-Dawley (SD) rats aged 12 weeks, which were purchased from the Southern Medical University (Guangzhou, China), were randomly divided into the Sham group and the BOO group. For induction of BOO, the rat’s proximal urethra tissues were first exposed by introducing a midline suprapubic incision under anesthesia with isoflurane treatment. Subsequently, the proximal urethra of rats in the BOO group were laced with a 4–0 silk suture using a 1 mm metal spacer, followed by removal of the 1 mm metal spacer, and closure of the abdominal wound. The same surgical operations were done in the Sham group, except for the ligature of rat proximal urethra. All rats were sacrificed 14 days after the surgery, whose bladder tissues were collected and immediately stored in liquid nitrogen for further processing. All experiments involving rats were approved by the Experimental Animal Care and Usage Committee of the Six Affiliated Hospital of Guangzhou Medical University (Qingyuan People’s Hospital) (Qingyuan, China).

### Deep RNA Sequencing

Total RNA samples were extracted from rat bladder tissues using the Eastep Super Total RNA Extraction Kit (#LS1040; Promega) according to the manufacturer’s instructions. The profiling and quantitation of differentially expressed circular RNAs in rat bladder tissues were done via the RNA sequencing assay as previously described with minor modifications (Fu et al., 2017). Briefly, total RNA samples were incubated with DNase I (Promega), followed by removal of rRNA components using the Ribo-off rRNA Depletion Kit (#N406-01; Vazyme, Nanjing, and China) following the manufacturer’s instructions. Linear RNA molecules in total RNA samples were then deleted by treating with the RNase R. The resultant circular RNAs were fragmented and subjected to construction of RNA sequencing library via random priming using the Ultr II RNA Library Preparation Kit (#E7770S; NEB, United States) as instructed by the producer. Following end repair, dA-tailing, ligation of adaptors and uracil-specific excision, and the library was finally enriched by PCR method. After size selection, the library was then sequenced with an Illumina Hiseq X ten sequencing system.

### circRNA Characterization and Bioinformatics

Raw reads from deep sequencing were filtered to obtain clean reads, which were then mapped to reference genome using the BWA-MEM software (Zhang et al., 2019). Subsequently, the prediction of circular RNAs in rat bladder tissues were performed using the CIRI software based on the back-splice algorithm (Glazar et al., 2014; Gao et al., 2015). The differential expression of target circular RNAs were determined by comparing the TPM (Transcripts Per Million) values between groups. Significant differential expression of circular RNAs were defined by the combination of a fold change of >2 and a *p* value of <0.05. The tendency of circRNA differential expression in rat bladder tissues were further presented by hierarchical clustering using “pheatmap” R package and volcano plot.

### Protein Digestion and Peptide Labeling

The differentially expressed proteins in rat bladder tissues were comprehensively characterized by the iTRAQ (isobaric tags for relative and absolute quantitation) as previously reported with minor modifications (Gao et al., 2020). Briefly, total protein samples extracted from rat bladder tissues via acetone precipitation were quantitated by the Bradford method. About 300 ug rat bladder proteins from each group were mixed with 30 ul SDT buffer, boiled at 100°C for 5 min, mixed with 200 ul UA buffer (8 M Urea, and 150 mM TrisHCl pH8.0), and incubated with 50 mM IAA for 30 min in darkness. Subsequently, rat bladder proteins were digested by overnight incubation with 40 µl Trypsin solution (4 µg Trypsin dissolved in 40 µl Dissolution buffer) at 37°C for 17–18 h. After centrifuge at 14000 g for 10 min, the collected peptides were quantitated by measuring the OD280 values. Approximately 100 ug rat bladder peptides in each group were subjected to labeling with the iTRAQ Reagent-8 plex Multiplex Kit (AB SCIEX) following the manufacturer’s instructions.

### Liquid Chromatography Separation

Before mass spectrometry, rat bladder peptides were first separated by liquid chromatography using a LC-20AT HPLC system (Shimadzu, Japan), equipped with a Gemini-NX C18 column (4.6 mm × 250 mm; 5 µm 110 Å Phenomenex; PN: 00G-4454-E0). Ammonium formate (20 mM; pH 10) was used as the Buffer solution A, and 80% acetonitrile (ACN) containing 20 mM Ammonium formate (pH 10) was applied as the Buffer solution B. Peptides powders were resuspended in Buffer solution A, centrifuged at 12,000 rpm for 20 min, and loaded onto to the C18 column, which were eluted using the following settings: 5% Buffer B, for 5 min; 15% Buffer B, for 25 min; 38% Buffer B, for 5 min; 90% Buffer B, for 10 min; 5% Buffer B, for another 10 min, under the flow rate of 0.8 ml/min. The collected peptides were lyophilized for mass spectrometry.

### LC-MS/MS

Separated rat bladder peptides were subsequently to LC-MS/MS (Liquid Chromatography-Tandem Mass Spectrometry) analysis using the AB SCIEX Triple-TOF 5600 mass spectrometer (AB SCIEX). Briefly, labelled rat bladder peptides were loaded onto the nano-LC HPLC system equipped with an EASY C18 column (75 µm * 100 mm; 3 μm; Thermo Fishier Scientific). Buffer A used for LC was 0.1% FA (formic acid), Buffer B was 0.1% FA solution containing 84% ACN, and the flow rate was set as 250 nl/min. The concentration gradient of Buffer B during the elution was as follows: 0%, 100 min; 0%–35%, 8 min; 35%–100%, 12 min. Subsequently, the separate peptides were subjected to mass spectrometric analysis using the AB SCIEX Triple-TOF 5600 system. The time scale for MS was 120 min, which was carried out in the positive ion mode using the following settings: precursor ion scanning range: 300–1800 m/z; MS1 resolution: 70,000 at m/z 200; AGC target: 3e6; Number of scan ranges: 1; Dynamic exclusion: 40.0 s. Ten MS2 scans were performed after each full scan, and the MS2 Activation Type was HCD, using an isolation window of 2 m/z, an MS2 resolution of 17,500 at m/z 200, a normalized collision energy of 30 eV and an Underfill ratio of 0.1%.

### Bioinformatic Analysis of Proteomic Data

Raw data from LC-MS/MS in the form of wiff files were analyzed using the ProteinPilot (AB SCIEX; Version: 4.5) for protein identification and quantitation, which was searched against the UniProt proteome dataset (species: rat; downloaded on May 31, 2017) that containing 36,663 entries. Unique peptides were characterized using an FDR (false discovery rate) of <0.05 using Benjamini-Hochberg method. Significantly differentially expressed proteins were defined by the combination of fold change (>1.5) and *p* value (<0.05). The R package “pheatmap” was used to perform the hierarchical clustering of differentially expressed proteins. Functional categorization of differentially expressed proteins based on GO (gene ontology) and KEGG (Kyoto Encyclopedia of Genes and Genomes) databases were done using the DAVID (Database for Annotation, Visualization and Integrated Discovery) website (https://david.ncifcrf.gov).

### Prediction of Protein-Encoding circRNAs

Bioinformatic prediction of protein-encoding circular RNAs was carried out in this study through selection of the existence of ORF (open reading frame) using the GETORF software (http://emboss.bioinformatics.nl/cgi-bin/emboss/getorf). Specifically, the predicted ORF for circRNA-encoded proteins should range over the junction sites of target circRNAs. The sizes of circRNA-encoded proteins must be more than 100 amino acid residues. In case of multiple ORFs predicted in single circular RNA, the one with the least amino acid residues was selected for the circRNA-encoded protein.

### Quantitative RT-PCR

Total RNA samples were extracted from rat bladder tissues as introduced above. The concentrations of RNA samples were determined by measuring the OD260 with a Thermo NanoDrop 2000 spectrometer. Approximately 1 ug RNA from each sample were used for the synthesis of cDNA by reverse transcription using the EasyScript M-MLV Reverse Transcriptase (#AE101-02; Transgen, Beijing, and China) following the producer’s instructions. The relative expression levels of circRNAs were then measured via the real-time quantitative PCR method using the ransStart Green qPCR SuperMix kit (#AQ101-01; Transgen, Beijing, and China) according to the manufacturer’s instructions. Primers used for quantitative PCR assay were listed in Table 1.

**TABLE 1:** The information of primers used for quantitative PCR method.

Gene ID	Primer sequence (5′-3′)	Product length (bp)
R-chr14:44496991|44498906-F	AGG​AAC​TGG​ATT​ATG​AAC​GGA​TTC​A	203
R-chr14:44496991|44498906-R	AGC​TAT​CTT​CTT​ATC​AGT​CAC​TGT​AT	
R-chr1:259396273|259437470-F	CTT​CCT​CTC​CCA​CAA​GCA​GG	215
R-chr1:259396273|259437470-R	CAT​CCC​GCC​AAG​GCA​TTT​TC	
R-chr9:81258380|81275269-F	ATA​GCC​CAC​TCC​TTC​CCC​AT	209
R-chr9:81258380|81275269-R	CAT​GGA​GCA​CCT​GGA​GAA​CG	
R-chr13:51600243|51609980-F	GGA​AGG​TTA​CAA​AGC​ACA​CCA​C	226
R-chr13:51600243|51609980-R	TCA​ACA​AGC​TCT​CGA​TGC​CT	
R-chr1:141877382|141880461-F	CGC​AGA​GTC​GAT​GGT​GAC​C	201
R-chr1:141877382|141880461-R	GGC​TCA​TTG​ACG​ACA​TGG​TG	
R-chrX:52581092|52765991-F	TTG​CTG​TTG​GAG​GTA​CCT​GC	212
R-chrX:52581092|52765991-R	TCC​CCA​GTT​GCA​TTC​AGT​GT	
R-chr19:39176540|39195019-F	GCC​GTG​TCA​TAG​CTA​CCC​TC	188
R-chr19:39176540|39195019-R	GAC​CTT​CCT​GTC​CTT​GGT​GG	

### Statistical Analysis

SPSS 20.0 were used for analysis. The expression values of circRNAs in quantitative PCR assay were expressed as mean ± SE (standard error). Mann-Whitney U test was used for comparison between groups. A *p* value of <0.05 was considered to be significant.

## Results

### Alterations of circRNA Profiles in Rat BOO Tissues

**FIGURE 1:**
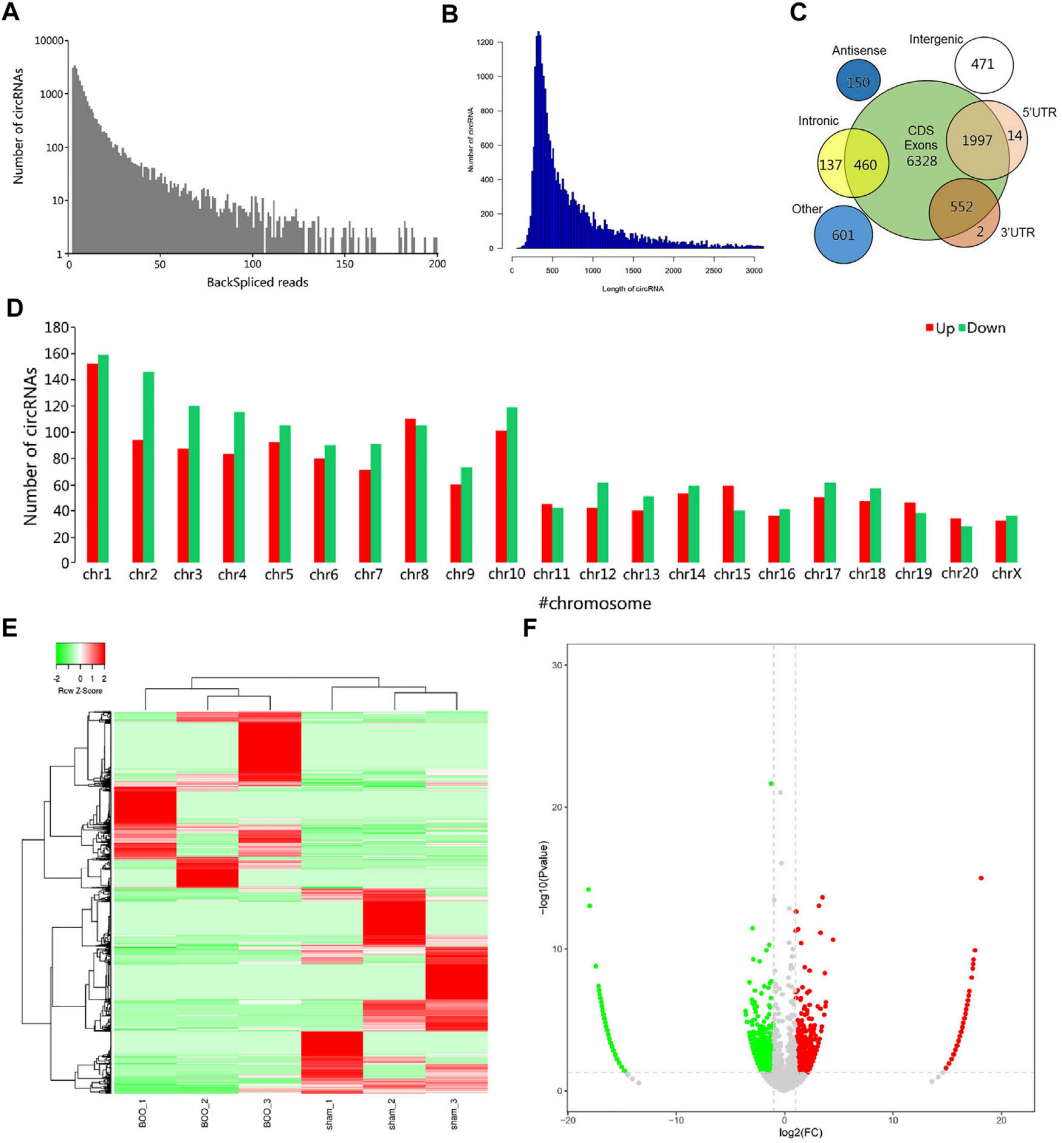
Characterization of differentially expressed circRNAs in rat BOO model. **(A)** Frequency distribution of circRNAs expression in rat BOO model based on backspliced reads. X axis: the range of total backspliced reads of circRNAs. Y axis: the frequency of circRNAs. **(B)** Frequency distribution of circRNAs in rat BOO model based on circRNA length. X axis: the range of circRNAs length; Y-axis: the frequency of circRNAs. **(C)** The numbers of circRNAs in rat bladder tissues with different genomic origins. **(D)** Numbers of circRNAs encoded by different rat chromosomes. The up- and down-regulated circRNAs in the rat BOO model were shown in red and green bars respectively. **(E)** Hierarchical clustering of differential circRNA expression in the rat BOO model. Increased and decreased expression of circRNAs were indicated by red and green lines respectively. **(F)** A volcano plot showing the differential expression of circRNAs in the rat BOO model. CircRNAs with elevated and reduced expression in the rat BOO model were shown by red and green spots respectively.

To explore the association of circRNA-encoded proteins with BOO pathogenesis, we established the rat BOO model as described in the Material and Method section, and then performed a large-scale identification of circRNAs differentially expressed in the rat BOO model by deep sequencing method (Figure 1A). In our RNA sequencing assay, we found that the lengths of circRNAs identified in rat bladder tissues range from 100 to over 3,000 bp, and the greatest enrichment of circRNAs length near 400 bp was observed in the circRNA profile in the rat bladder tissues (Figure 1B). Also, the circRNAs in rat bladder tissues were shown to be encoded by different gene regions, including CDS (coding sequences), 5′-UTR (untranslated region), introns, 3′-UTR, intronic, antisense, and intergenic regions (Figure 1C). The majority of circRNAs identified in rat bladder tissues were encoded by the CDS regions (Figure 1C). Totally, 3,051 circRNAs were found to be differentially expressed in rat BOO tissues compared with the Sham group, including 1,414 up-regulated, and 1,637 down-regulated circRNAs in the BOO group (Supplementary Table S1; Figures 1D–F). Genome distribution analysis showed that differentially expressed circRNAs in rat bladder tissues of the BOO group were originated from all rat chromosomes, especially the #1–10 chromosomes (Figure 1D). The significant alterations of circRNA profiles in the BOO group were further presented by the hierarchical clustering and volcano plot (Figures 1E,F). The great changes of circRNA expression in the rat BOO model suggested the potential roles of circRNAs in BOO development and progression.

### Differentially Expressed Proteins in Rat BOO Model

**FIGURE 2:**
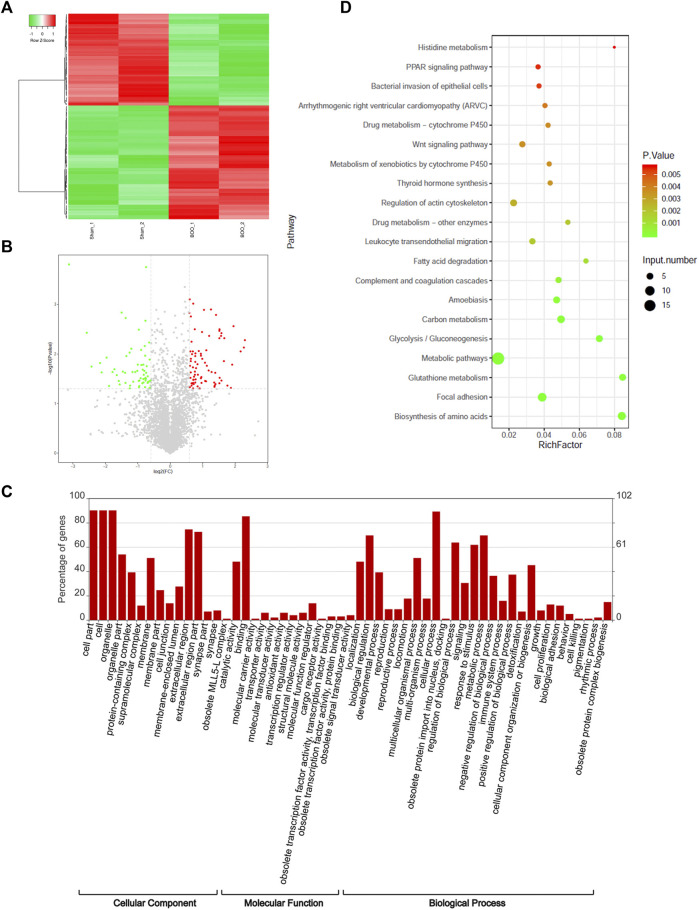
Proteomic identification of differentially expressed proteins in rat BOO model. **(A)** Hierarchical clustering of proteins differentially expressed in the bladder tissues of rat BOO model. Elevation and decreases of protein expression compared with the Sham group were present as red and green lines respectively. **(B)** The volcano plot displaying differentially expressed proteins in the bladder tissues of the rat BOO model. Red and green spots indicate up-regulated and down-regulated proteins respectively. **(C)** Functional categorization of proteins differentially expressed in the bladder tissues of the BOO rats. The enrichments of differentially expressed proteins in GO cellular components, molecular functions and biological processes were performed separately. **(D)** Functional categorization of differentially expressed proteins in rat BOO model based on KEGG signaling pathways. The enrichment significances were indicated by spot colors and protein numbers were indicated by the spot diameters.

The differential expression of proteins in the bladder tissues of rat BOO model were subsequently characterized by the iTRAQ quantitative proteomic method, using the Sham group as the control. In total, 85 proteins were shown to be differentially expressed in the BOO group in comparison with the control group, including 45 up-regulated and 40 decreased proteins in the rat BOO model (Figures 2A,B; Supplementary Table S2). Through functional categorization based on GO cellular components, differentially expressed proteins in rat BOO model were mainly distributed in protein-containing complexes, membranes, cell junctions, extracellular regions, and obsolete MLL5 (Mixed lineage leukemia 5)-L complex and synapse part (Figure 2C). Based on their molecular functions, differentially expressed proteins in rat bladder tissues were mainly associated with molecular carries, transporter, antioxidant, transcription regulator, structural molecule, cargo receptor, and obsolete signal transducer activities (Figure 2C).

Furthermore, our categorization based on the GO biological processes showed that proteins differentially expressed in rat BOO model were significantly enriched in various biological processes, including the development, reproduction, locomotion, obsolete protein import into the nucleus, signaling, responses to stimulus, metabolism, immune system, detoxification, cell proliferation, biological adhesion, cell killing, pigmentation, and rhythmic processes and obsolete protein complex biogenesis (Figure 2C). Moreover, these differentially expression proteins were also enriched in multiple KEGG signaling pathways, such as histidine metabolism, PPAR (peroxisome proliferator-activated receptor) signaling, bacterial invasion of epithelial cells, Arrhythmogenic right ventricular cardiomyopathy (ARVC), drug metabolism via cytochrome P450, Wnt (wingless/integrated) signaling, thyroid hormone synthesis, regulation of actin cytoskeleton, leukocyte transendothelial migration, fatty acid degradation, complement and coagulation cascades, amoebiasis, carbon metabolism, glycolysis, and other metabolic pathways (Figure 2D). These investigations showed that the pathogenic development of BOO was associated with various biological processes and signaling cascades.

### Characterization of circRNA-Encoded Novel Proteins in Rat BOO Model

**FIGURE 3:**
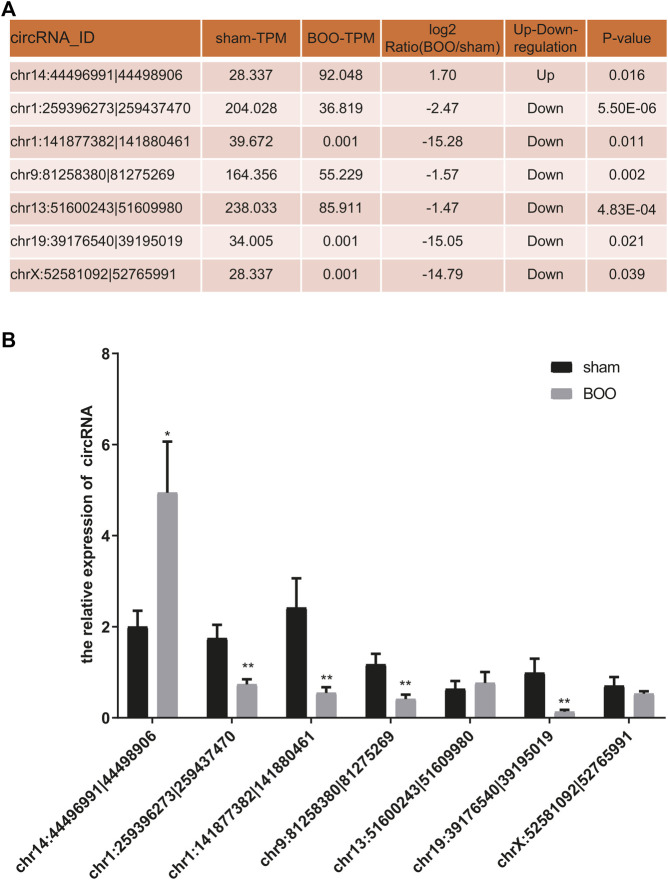
Validation of protein-encoding circRNA expression in rat BOO model. **(A)** The list of predicted protein-encoding circRNAs selected for validation and their expressional alterations in rat BOO model shown by RNA sequencing. The expressional ratio between the Sham and BOO groups, as well as the statistical significances (*p* values), during the RNA sequencing were shown in the table. **(B)** Relative expressional levels of representative protein-encoding circRNAs in the rat BOO model. The expression of circRNAs in rat BOO model (*n* = 7) were measured by quantitative RT-PCR method, compared with the Sham group (*n* = 8). *p < 0.05; **p < 0.01. Error bar indicated standard error. Mann-Whitney U test was used for comparison between groups.

For characterization of circRNA-encoded protein associated with BOO pathogenesis, we first bioinformatically predicted the differentially expressed circRNAs (Figure 1) that contains ORF using the GERORF software. Subsequently, above-mentioned iTRAQ quantitative proteomic data were analyzed combined with the RNA sequencing data and ORF prediction results, and we found that the proteins encoded by 21 ORF-containing circRNAs were identified by our proteomic analysis, which exhibited consistent expressional alterations in both the circRNA and protein levels as shown by RNA sequencing and quantitative proteomic analyses respectively (Supplementary Table S3). Among them, the expression of 7 predicted protein-encoding circRNAs in the rat BOO model were further verified by quantitative RT-PCR, and we found that the expression of five ORF-containing circRNAs in the rat BOO model were consistent with the RNA sequencing results (Figures 3A,B). Among them, one protein-encoding circRNA (chr14:44496991|44498906) was up-regulated and four other protein-encoding circRNAs were down-regulated in the bladder tissues of rat BOO model compared with the Sham group, including chr1:259396273|259437470, chr1:141877382|141880461, chr9:81258380|81275269, and chr19:39176540|39195019 (Figures 3A,B).

**FIGURE 4:**
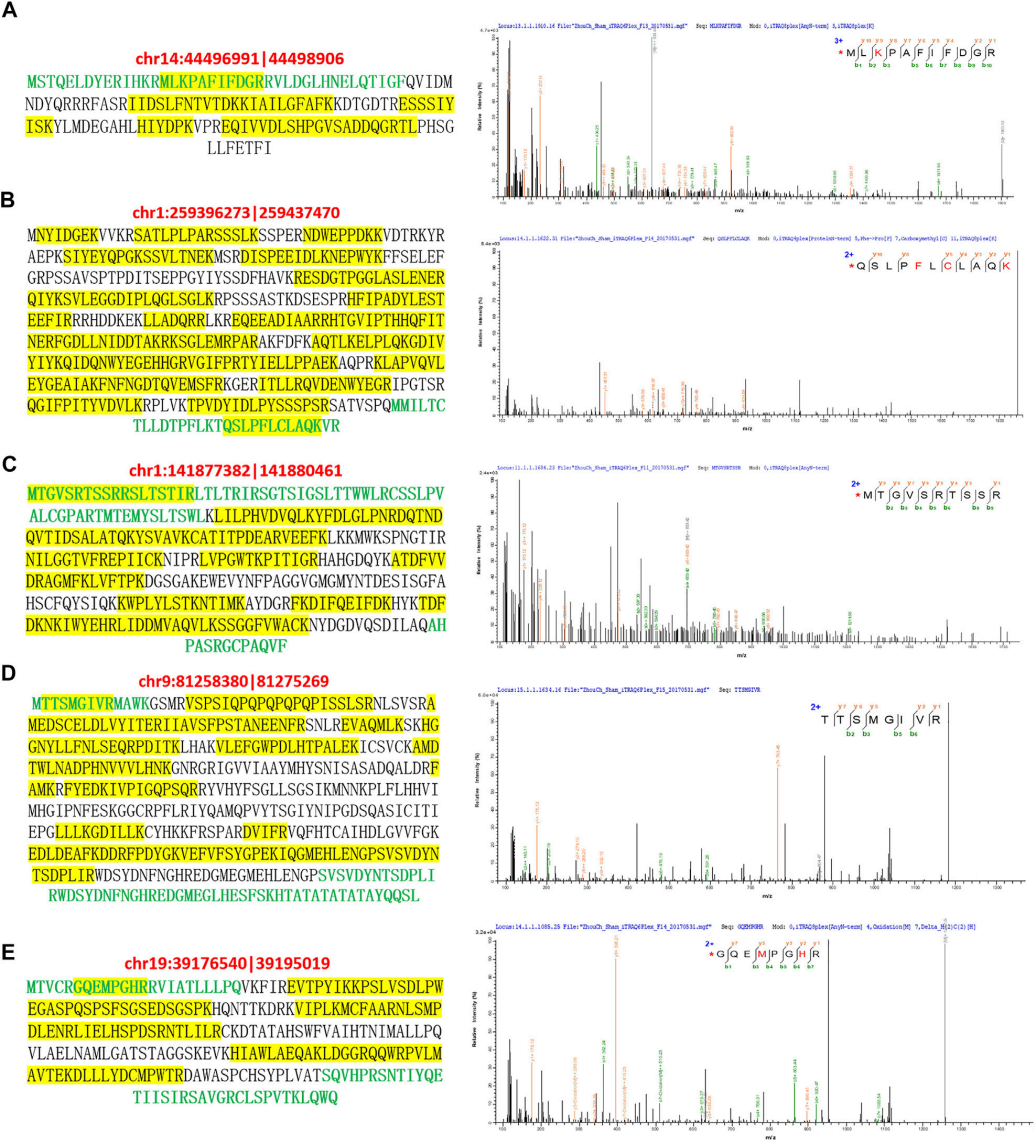
Mass spectrometric identification of circRNA-encoded proteins in rat BOO model. The amino acid sequences of novel proteins encoded by circRNAs chr14:44496991|44498906 **(A)**, chr1:259396273|259437470 **(B)**, chr1:141877382| 141880461 **(C)**, chr9:81258380|81275269 **(D)**, and chr19:39176540|39195019 **(E)** were listed in the left. Unique amino acid sequences encoded by circRNAs were shown in green, and peptides identified by mass spectrometry were highlighted by yellow background. The mass spectrometric identification of unique amino acid sequences in these five circRNA-encoded proteins were shown in the right.

Finally, the expression of novel proteins encoded by these five differentially expressed circRNAs were further validated by analyzing our proteomic data (Figure 4). For instance, the novel protein encoded by the circRNA chr14:44496991|44498906 were predicted to possess a unique amino acid sequence as shown by green characters, which was identified by mass spectrometry in our iTRAQ quantitative proteomics (Figure 4A). Besides, multiple other peptides in this novel protein encoded by circRNA chr14:44496991|44498906 were identified by our mass spectrometry, which were shown by yellow background in Figure 4A. In addition, the unique amino acid sequences of other four novel proteins encoded by above-mentioned circRNAs were also identified by mass spectrometry during our proteomic analysis (Figures 4B–E). These mass spectrometric results solidly validated the expression of circRNA-encoded proteins in the bladder tissues of rat BOO model.

## Discussion

The pathogenesis and progression of BOO was previously known to be closely associated with multiple anatomic, functional and also neurologic factors which contributed to urothelium abnormality such as intrafascicular fibrosis (King and Goldman, 2014; Meier and Padmanabhan, 2016; Fusco et al., 2018), however, the molecular events driving the pathogenic alterations in urothelium during BOO development still remains poorly understood. In recent years, circRNAs were widely expressed in various human organs and tissues which were prevalently involved in pathogenesis of diseases, but its expression and linkage with BOO development are still unclear (Kristensen et al., 2019; Li et al., 2021). In the present study, we established the rat BOO model through the partial bladder outlet obstruction surgery and characterized circRNAs differentially expressed in bladder tissues of rat BOO model by deep RNA sequencing method, which revealed great alterations of circRNA profiles possibly associated with BOO development. One major mechanism of circRNA functioning in development and disease development was mediated by their capability of encoding novel proteins (Zheng et al., 2019; Jiang et al., 2021). To explore the possibility of circRNA-encoded proteins in BOO pathogenesis, we performed an iTRAQ quantitative proteomic analysis of differentially expressed proteins in rat BOO model, and which revealed great changes of proteome enriched in various biological processes and signaling pathways. More importantly, the combined analysis of RNA sequencing and proteomics discovered multiple novel circRNA-encoded proteins which were significantly differentially expressed in rat BOO model. These investigations showed for the first time the potential functions of circRNA and circRNA-encoded proteins in BOO pathogenesis.

During the past decade, the epigenetic landscapes underlying human disorder development have been substantially broadened by the discovery of significant pathogenic effects of non-coding RNA (Ding et al., 2021; Laneve and Caffarelli, 2020). Among them, microRNAs, which are commonly composed of 18–25 nucleotides, performs their regulatory roles mainly through repressing target protein translation, or accelerating mRNA degradation (Bartel, 2018). One recent report showed that microRNA profiles were greatly altered in the rat BOO model, which revealed important roles of miR-29 and other regulatory microRNAs in organ deformation, and bladder remodeling linked with BOO development (Ekman et al., 2016). As introduced above, the circular RNAs have recently been established as another key kind of non-coding RNAs due to their regulation of target gene transcription and association with microRNAs or proteins (Li X. et al., 2018). In term of bladder biology and pathogenesis, recent progresses were mainly focused on the roles of circRNAs in regulating bladder cancer development (Li M. et al., 2018; Xie et al., 2018; Lu et al., 2019; Liu et al., 2020). Here in this study, we showed that more than 3,000 circRNAs were differentially expressed in the bladder tissues of our rat BOO model, and both the numbers of up-regulated and down-regulated circRNAs were more than 1,400, which is the first characterization of circRNAs profiles underlying the BOO pathogenesis. These results suggested the potent functions of circRNAs during BOO initiation and development, which could also serve as a basis for elucidating the non-coding RNA-mediated molecular mechanisms underlying BOO and other urologic conditions.

Based on previous research, the prevalent involvements of circRNAs in human physiology and pathogenesis have been mainly attributed to their modulation of functional gene expression and interrupting microRNA functions (Kristensen et al., 2019; Li et al., 2021). However, the discoveries of novel proteins specifically encoded by circRNAs in recent years unveiled another layer of molecular mechanisms underlying circRNA-regulated physiological processes and disease development (Begum et al., 2018; Xia et al., 2019; Zheng et al., 2019; Pan et al., 2020; Jiang et al., 2021). As introduced above, the novel protein SHPRH-146aa, which was encoded by the circ-SHPRH, regulates glioblastoma tumorigenicity via stabilizing the full-length SHPRH (SNF2 histone linker PHD RING helicase) protein by inhibiting its ubiquitin-mediated degradation (Begum et al., 2018; Wu et al., 2020). To address whether circRNA-encoded novel proteins are expressed in bladder tissues and their possible involvements in BOO pathogenesis, we also performed a quantitative proteomic analysis of bladder tissues from rat BOO model. Interestingly, the novel proteins predicted to be encoded by as many as 21 circRNAs, whose expression were significantly altered in rat BOO model, were identified in our proteomic analysis and showed protein abundance changes consistent with their corresponding circRNA levels. More importantly, the expressional alterations of five of these protein-encoding circRNAs in rat BOO model were further validated by quantitative RT-PCR. These results provided the first and strong evidence of the existence and expressional changes of circRNA-encoded novel proteins in BOO development. It should be noted that their biological roles in bladder physiology and BOO pathogenesis deserve further investigations.

Our quantitative proteomics also revealed great changes of functional proteins in rat BOO model, which were linked with various biological processes and signaling cascades. For instance, differentially expressed protein in rat BOO model were found here to be significantly enriched in the PPAR signaling pathway. Previous reports showed that the abnormal activation of the PPARγ and related signaling events contributed to the overexpression of retinoid X receptors (RXRs) during luminal bladder tumor initiation and progression (Rochel et al., 2019). The great alterations of PPAR signaling proteins in rat BOO model shown in this study indicated the potential roles of PPAR signaling in BOO pathogenesis. Furthermore, the WNT signaling pathway performed essential roles in regulating urothelial development and maintaining the homeostasis of urothelial tissues, which also was identified as key signaling hubs driving the urothelial tumorigenesis, and metastasis and relapse because of its modulating the urothelial cancer stem cell activities (Garg and Maurya, 2019). In this study, we also characterized a set of WNT signaling proteins that were differentially expressed in the rat BOO model, which pointed to the potential implication of WNT-related signaling in BOO pathogenesis. More importantly, cellular signaling such as the PPAR and the WNT pathways could be prevalently regulated by circRNAs (Guo et al., 2018; Huang et al., 2020), and the interactions between circRNA and these signaling cascades during BOO development might be an interesting question for future analysis. In addition, the possible roles of circRNA-encoded novel proteins in signaling events underlying BOO pathogenesis also deserve further investigations.

In summary, we performed the first characterization of circRNA and circRNA-encoded novel proteins associated with BOO development by the combination of RNA sequencing and quantitative proteomics, which revealed great alterations of circRNA and proteomic profiles in rat BOO model, and as well as the existence and differential expression of circRNA-encoded proteins. These results suggested potential new roles of circRNAs in BOO development possibly mediated by signaling pathways induced by circRNA-encoded novel proteins, and further functional research would provide new insights into the molecular mechanism underlying the pathogenesis of BOO and other urologic disorders.

## Data Availability

The datasets presented in this study can be found in online repositories. The mass spectrometry proteomics data are available via ProteomeXchange with identifier PXD029337. The RNA sequencing data are available via SRA data (BioProject accession number: PRJNA772547.
